# MicroRNA-101 is a potential prognostic indicator of laryngeal squamous cell carcinoma and modulates CDK8

**DOI:** 10.1186/s12967-015-0626-6

**Published:** 2015-08-19

**Authors:** MingHua Li, LinLi Tian, Hui Ren, XiaoXue Chen, Yu Wang, JingChun Ge, ShuLiang Wu, YaNan Sun, Ming Liu, Hui Xiao

**Affiliations:** Services of Head and Neck Surgery, Department of Otolaryngology-Head and Neck Surgery, The Second Affiliated Hospital of Harbin Medical University, No. 148, Bao jian Road, Harbin, 150081 People’s Republic of China; Services of Laryngology, Department of Otolaryngology-Head and Neck Surgery, The Second Affiliated Hospital of Harbin Medical University, No. 148, Bao jian Road, Harbin, 150081 People’s Republic of China; The First Clinical Hospital Affiliated to Harbin Medical University, Harbin, 150001 People’s Republic of China; The Human Anatomy and Histoembryology Department, Harbin Medical University, Harbin, 150081 People’s Republic of China

**Keywords:** Apoptosis, CDK8, Laryngeal squamous cell carcinoma, MiR-101, Proliferation

## Abstract

**Background:**

Various microRNAs (miRNAs) negatively modulate genes that are involved in cellular proliferation, differentiation, invasion, and apoptosis. In many types of cancer, the expression profiles of these miRNAs are altered. Recently, miR-101 was identified as a tumour suppressor and was found to be expressed at low levels in various types of tumours, including prostate, breast, endometrium, and bladder cancers. However, the function(s) of miR-101 in laryngeal carcinoma remain unknown.

**Methods:**

The expression levels of miR-101 in laryngeal squamous cell carcinoma (LSCC) tissues and cells were detected by qPCR. Cell proliferation, migration, cell cycle, and apoptosis assay were applied to assess the function(s) of miR-101 in vitro. Nude mice subcutaneous tumour model was used to perform in vivo study. Moreover, we identified Cyclin-dependent kinase 8 (CDK8) as the target of miR-101 by a luciferase assay. The possible downstream effectors of CDK8 were investigated in Wnt/β-catenin signaling pathway. Changes of CDK8, β-catenin, and cyclin D1 protein levels were analyzed by western blotting and immunohistochemical staining. The prognostic effect of miR-101 was evaluated using the Kaplan–Meier method.

**Results:**

Expression of miR-101 was down-regulated in the LSCC tissues compared with the adjacent normal tissues. Furthermore, downregulation of miR-101 correlated with T3–4 tumour grade, lymph node metastasis, and an advanced clinical stage in the LSCC patients examined (P < 0.05). The low level of miR-101 expression was associated with poor prognosis (P < 0.05). CDK8 was identified as the target gene of miR-101 by luciferase reporter assay. Moreover, we showed that up-regulation of miR-101 expression suppressed humen LSCC Hep-2 cells proliferation and migration, and induced cell-cycle arrest. Increased expression of miR-101 induced cells apoptosis both in vitro and in vivo. Correspondingly, exogenous expression of miR-101 significantly reduced the growth of tumour in a LSCC xenograft model. Furthermore, the miR-101 level was inversely correlated with levels of CDK8, β-catenin, and cyclin D1 in western blotting assay and immunohistochemical staining assay.

**Conclusions:**

These results indicate that miR-101 is a potent tumour repressor that directly represses CDK8 expression. Thus, detection and targeting of miR-101 may represent a novel diagnostic and therapeutic strategy for LSCC patients.

**Electronic supplementary material:**

The online version of this article (doi:10.1186/s12967-015-0626-6) contains supplementary material, which is available to authorized users.

## Background

Laryngeal malignancies are the second most common cancers of the head and neck, and more than 95 % of cases worldwide are diagnosed as laryngeal squamous cell carcinoma (LSCC) [1–3]. The incidence of LSCC is also higher in males than in females. According to the American Cancer Society, 12,720 new cases and 3,600 deaths due to LSCC were estimated for the United States in 2010 [4]. Treatments for cancer of the larynx include surgery, radiotherapy, chemotherapy, or a comprehensive therapy approach. Despite improvements in diagnostic and therapeutic techniques, the 5-year survival rates for patients with LSCC have not increased over the last 20 years [5]. Most patients diagnosed with advanced-stage laryngeal cancer die of recurrence and/or metastasis. Thus, a better understanding of the molecular mechanisms involved in the carcinogenesis of LSCC would facilitate the development of much needed targeted therapies.

MicroRNAs (miRNAs) are a family of small, single-stranded, noncoding RNAs that bind target genes to negatively regulate their expression by repressing translation and/or by causing mRNA degradation [6]. An estimated 30 % of human mRNAs possess conserved miRNA-binding sites [7]. A number of studies have shown that miRNAs contribute to many basic cellular functions including proliferation, differentiation and death [8–10]. Moreover, miRNAs have been found to play a critical role in tumourigenesis, either by serving as oncogenes or tumour suppressor genes [11–13]. Accordingly, dysfunction of specific miRNAs has been linked to tumourigenesis and cancer progression. [14, 15]. Thus, it is possible that some of these miRNAs may represent ideal targets for predicting and treating various cancers [16]. Recent studies have shown that some miRNAs are aberrantly expressed and involved in the regulation of the malignant behavior of LSCC, such as cell invasion, metastasis and apoptosis [17–20]. These miRNAs may contribute to the elucidation of the molecular mechanisms involved in LSCC pathogenesis and the development of effective diagnostic methods and therapeutic strategies. A miRNA profiling using microarray analysis identified hsa-miR-101 was down-regulated in head and neck cancers samples compared to normal samples [21], which interested us in the role miR-101 played in LSCC.

MiR-101 is a highly conserved miRNA, whose encoding genes locate at chromosome 1p31.3 and chromosome 9p24.1 and undergo abnormal deletions in several malignant cells [22]. MiR-101 has been found to be frequently down-regulated in many types of tumours, including colon [23], prostate [24, 25], lung [26, 27], gastric [28], endometrium [29], breast [30], bladder [31, 32], melanoma [33], and head and neck squamous cell carcinomas [34]. Furthermore, miR-101 has been found to function as a tumour suppressor. Ectopic expression of miR-101 significantly inhibits cell proliferation, migration, and invasion by regulating genes involved in these processes (e.g., *COX*-*2* [23, 24, 28], *EZH2* [25, 27–29, 33, 34], *Mcl*-*1* [28, 29], *Fos* [29], *Stathmin1* [30] and *c*-*Met* [32]). However, the function(s) of miR-101 in laryngeal carcinoma remain unknown.

CDK8 is a member of the CDK family, involved in transcriptional regulation from yeast to mammals [35, 36]. Currently, mechanisms for the regulation of CDK8 activity are not fully known. Most of what is known about CDK8 results from its facultative association with the Mediator complex, but functions alone are also likely [37–39]. A growing body of research provides unequivocal evidence for CDK8 as coactivator in several transcriptional programs. For example, CDK8 plays an important regulatory role in biological processes at the transcription level in the Wnt/β-catenin signaling pathway and it is proposed to be a proto-oncogene in human colon cancer [40–42]. The computer sequence analysis (TargetScan and miRDB [7, 43]) suggested that the 3′ untranslated region (UTR) of CDK8 mRNA might represent a target of miR-101.

The purpose of this study is to explore the role of miR-101 in LSCC cell proliferation, invasion, apoptosis and cell cycle regulation. Another goal is to investigate the underlying mechanism of miR-101 functions in LSCC. In this study, we found that miR-101 was down-regulated in LSCC cell lines and tissues. And miR-101 inhibited the tumourigenesis progression through the regulation of Wnt/β-catenin signaling pathway by targeting CDK8 directly in LSCC. Therefore, our findings demonstrate the role of tumour suppressor of miR-101 in LSCC progression and indicate that miR-101 might serve as a prognostic and therapeutic target for LSCC.

## Methods

### Samples

The patients who might have died for reasons other than the disease itself were excluded from the study. All of the 80 patients who underwent partial or total laryngectomy at the Department of Otorhinolaryngology in the Second Affiliated Hospital of Harbin Medical University between 2008 and 2009 were diagnosed with primary laryngeal squamous cell carcinoma by the pathologist. The tumour specimen was taken from the center of tumour tissue. And the center of tumor tissue was the enrichment area of LSCC cells, which had been conformed by pathology. The adjacent normal tissues we used in this study were the tissues about 1.5–2 cm from the tumour border diagnosed without precancerous or cancerous lesion in pathology. Pairs of LSCC tissues and adjacent normal tissues were collected during surgery and were immediately snap-frozen in liquid N_2_ for 5 min. Samples were then stored at −80 °C until processed. None of the enrolled patients received any preoperative therapy, and written informed consent and clinicopathological data were obtained from all of the patients. The research protocol used was approved by the Ethics Committee of Harbin Medical University. (Approval number: 2013-041).

### MiRNA expression assay

Total RNA was extracted from cells and tissues using Trizol reagent (Invitrogen, Carlsbad, CA, USA) according to the manufacturer’s protocol. The RNA samples were then reverse transcribed into cDNA using an All-in-One™ miRNA Q-PCR Detection Kit (Genecopoeia, Germantown, MD, USA). Real-time PCR was performed using a SYBR-Green Master Mix (ABI, Foster, CA, USA) and a 7500 Fast Real-Time PCR system (Applied Bio-System, Foster City, CA). Reaction conditions included: 95 °C for 10 min, followed by 40 cycles of 95 °C for 10 s, 57 °C for 20 s, and 72 °C for 15 s. Expression data were calculated from the CT values and were normalized to expression of the human U6 gene in each sample using the 2^−ΔCt^ method [44, 45]. Forward and reverse primers were used to detect has-miR-101 (5′-GAGGGGTACAGTACTGTGATA-3′ and 5′-TGCGTGTCGTGGAGTC-3′, respectively) and hsRNA-U6 (5′-GCTTCGGCAGCACATATACTAAAAT-3′ and 5′-CGCTTCACGAATTTGCGTGTCAT-3′, respectively) (Genechem, Shanghai, China). Each sample was measured in triplicate.

### Luciferase reporter assays

The human wild CDK8 3′ untranslated region (UTR) (base 92–99, 5′-GUACUGUA-3′) was amplified and cloned into the multiple cloning sites in a psi-CHECKTM-2 luciferase miRNA expression reporter vector (Promega). Site-directed mutagenesis of the miR-101 target site in the CDK8-3′-UTR (5′-AUGCGGCA-3′) was used as a negative control and termed CDK8-3′UTR mutant. The primers selected were as follows: CDK8-3′UTR, 5′-ATGCACTGTTGCGAATGCTG-3′ (forward) and 5′-AATGCTTGCCCCTAGCACAT-3′ (reverse); CDK8-3′UTR mutant, 5′-GAGAATATGCGGCAACAACC-3′ (forward) and 5′-GGTTGTTGCCGCATATTCTC-3′ (reverse). For the reporter assays, cells were transiently transfected in 24-well plates with luciferase reporter gene constructs and has-miR-101, or an antagomir that was designed to target endogenous has-miR-101, using Lipofectamine 2000 (Invitrogen). Firefly and Renilla luciferase activities for each transfected well were measured 48 h after transfection using dual luciferase assay reagents (Promega). Three independent transfection experiments were performed in triplicate for each plasmid construct.

### Cell growth, transductions, and lentiviral production

The human LSCC cell line, Hep-2 (Cell Bank of Chinese Academy of Science, Shanghai, China), and the 16HBE cell line (Xiangfu Bio, Shanghai, China) were maintained in Dulbecco’s Modified Eagle’s Medium (DMEM; ThermoFisher Scientific, Waltham, MA) supplemented with 10 % fetal bovine serum (FBS) (Shenggong, Shanghai, China) at 37 °C under a humidified atmosphere containing 5 % CO_2_. Has-miR-101 and a green fluorescent protein (GFP) sequence were cloned into a recombinant lentivirus vector (Genechem, Shanghai, China) (Additional file 1: Fig. S1). Lentiviruses containing only the GFP cassette were used as a negative control. We named the lentivirus containing miR-101 sequence as miR-101 lentivirus and the cells treated with miR-101 lentivirus as the miR-101-treated group; lentivirus containing only the GFP cassette as GFP-lentivirus and the cells treated with GFP-lentivirus as the negative control group; the cells without any treatment as the blank control group. Briefly, cells were seeded in 6-well plates (1 × 10^5^ cells/well). After 12 h, 1 ml of complete medium containing lentivirus (10^8^ TU/ml) and polybrene (8 mg/ml) were added to each well according to the manufacturer’s protocol. Cells were incubated at 37 °C for 12 h, then were incubated in DMEM medium containing 10 % FBS and 1 % penicillin–streptomycin for an additional 24 h. Seventy-two hours after transduction, the infected cells were maintained in fresh DMEM and the mean percentage of GFP-positive cells present were calculated from three random fields-of-view (FOV) per well using a fluorescence microscope (IX70, Olympus, Japan) at 200× magnification.

### Animal experiments

Twenty-four healthy, 5-week-old, male BALB/c nude mice (~20 g each) were randomly divided into three groups (n = 8 per group). Each mouse received a subcutaneous injection of a Hep-2 cell suspension containing about 1 × 10^6^ cells (100 μl) into the dorsal scapula region. Tumour growth was subsequently measured twice a week and was calculated according to the following formula: ½ × length × width^2^. When the tumours reached a volume of ~0.5–0.6 cm^3^, the mice received various injections (injected into tumours) according to their treatment group. The mice in the treated group received an injection of miR-101 lentivirus (10^8^ TU/ml, 100 µl) once a week, the negative control mice received an injection of GFP-lentivirus (10^8^ TU/ml, 100 µl) once a week, and the blank control mice received an injection of 100 µl DMEM once a week. After 4 weeks of these injections, the mice were sacrificed under ether anesthesia and the tumours were dissected for further analysis.

During the experimental period, the mice had free access to food and water and were maintained in sterile micro-isolator cages under specific pathogen-free conditions at 27 ± 1 °C and 50 ± 10 % humidity. The mice were also exposed to a 10-h lights on/14-h lights off cycle. Moreover, all BALB/c nude mice were under ether anesthesia before they were sacrificed. All animal handling and experimental procedures were performed in accordance with the guidelines of the Care and Use of Laboratory Animals published by the China National Institution of Health to ensure the implementation of the animal welfare measures.

### Cell Counting Kit 8 (CCK8) cell proliferation assay

Cells were transfected and plated in 96-well plates (2 × 10^3^ cells/well). At various timepoints after transduction (0, 24, 48, 72, and 96 h), 10 μL of CCK8 reagent (C0038, Beyotime Inst Biotech, China) was added to each well according to the manufacturer’s protocol. After the plates were incubated at 37 °C for 4 h, optical density values at 450 nm were measured for each well using a microplate reader (Multiscan MK3; Thermo Labsystems, USA). The average value for each set of five replicate wells for each group was calculated. The percentage rate of cell growth was calculated using the following formula: (mean absorbance of the treatment group/mean absorbance of the control group) × 100.

### Cell migration assays

Seventy-two hours after transduction, 200 μL of serum-free medium containing 2 × 10^4^ cells from each group were added into the upper compartments of 24-well Boyden chambers (8 μm pore size) that had been coated with Matrigel (Becton–Dickinson Labware). In the lower compartment of each well, 1 ml of medium containing 10 % FBS served as a chemoattractant. After 24 h at 37 °C, the cells on the top side of the filters were carefully removed, while cells that migrated to the bottom side of the filters were fixed with 4 % paraformaldehyde and stained with haematoxylin and eosin (H&E). Stained cells were observed at 200× magnification and were counted in five randomly selected non-overlapping fields to provide an average number of migrated cells. Three independent experiments were performed.

### Cell cycle assays

Seventy-two hours after transduction, cells were harvested by trypsinization, washed twice using cold PBS and fixed in 70 % ethanol at 4 °C. After 2 h, the cells were washed with PBS, were treated with RNase A (50 μg/ml), and were stained with propidium iodide (PI) (25 μg/ml) at 37 °C. After 30 min, 2 × 10^5^ cells of each sample were analysed using a flow cytometer (FACS Calibur; Becton–Dickinson Immunocytometry Systems, San Jose, CA, USA). The distribution of cells among the cycle phases was determined using Modfit software (LT for Mac, V 3.0).

### Apoptosis assay

Cell apoptosis was assessed using an Annexin V-FITC and PI double-stain detection kit (Key Gen Biotech, Nanjing, China) according to the manufacturer’s protocol. Briefly, cells were harvested 72 h after transduction and were resuspended in Annexin-binding buffer at a concentration of 1 × 10^6^ cells/mL. Cells were then stained with Annexin V-FITC and PI at room temperature in the dark. After 15 min, the cells were analysed by flow cytometry. The experiments were repeated in triplicate.

Apoptotic cells in tumour sections were detected by terminal deoxynucleotidyl transferase dUTP nick end labelling (TUNEL) using an In Situ Cell Death Detection Kit (R&D, USA). Briefly, after routine deparaffinization, sections were digested with a proteinase K solution for 25 min, then were incubated with a blocking solution. After 15 min, the sections were incubated with 50 μL of a TUNEL reaction mixture for 60 min, then with an alkaline phosphatase antibody for 20 min. Diaminobenzidine (DAB) was used as a chromogen to enhance positive signals. The slices were also counterstained with haematoxylin. Sections were then dehydrated and mounted. All incubations were performed at 37 °C under a humidified atmosphere. Negative controls were prepared by treating the samples without terminal deoxynucleotidyl transferase (TdT). To quantitatively analyse the data, the percentage of TUNEL-positive cells per 200 tumour cells were averaged from 10 randomly-selected FOV per section using light microscopy at 400× magnification (Olympus, Tokyo, Japan).

### Western blotting

Cultured cells from the treated group, the negative control group, and the blank control group were harvested 72 h post- transduction and were incubated with cell lysis buffer on ice. After 30 min, the cell lysates were separated by sodium dodecyl sulfate–polyacrylamide gel electrophoresis (SDS-PAGE) in 10 % polyacrylamide gels and then were transferred to polyvinylidene fluoride (PVDF) membranes. Non-specific binding was blocked with a solution containing 5 % skim milk in Tris-buffered saline (TBS) containing 0.05 % Tween-20 (TBST). The membranes were then incubated with primary antibodies overnight at 4 °C. The primary antibodies used included rabbit anti-human CDK8 (1:200; Bioss, Beijing, China), rabbit anti-human β-catenin (1:200; Abbiotec, USA), and mouse anti-human cyclin D1 (1:200; Zhongshan Golden Bridge Biotechnology, Beijing, China). The membranes were subsequently washed with TBST and were incubated with species-appropriate horseradish peroxidase-conjugated secondary antibodies at 37 °C. After 1 h, the bands representing bound antibodies were quantified using Image J software (NIH, Bethesda, MD, USA). Detection of β-actin served as a loading control.

### Immunohistochemistry

Formalin-fixed, paraffin-embedded samples were sectioned (4 μm) sequentially. After deparaffinization and rehydration, the sections were treated with 0.3 % H_2_O_2_ to quench endogenous peroxidase activity and then were blocked with 10 % normal goat serum for 20 min. Antigen retrieval was performed using ethylene diamine tetraacetic acid (EDTA) (pH 8.0) at 100 °C for 20 min. Each section was incubated with the appropriate primary antibody overnight at 4 °C. After a second incubation step at 37 °C for 45 min, sections were incubated with secondary antibodies at room temperature. After 1 h, peroxidase signal was developed by incubating sections with diaminobenzidine tetrachloride for 10 min. The sections were also counterstained with haematoxylin. Negative control sections were incubated with PBS instead of primary antibody.

### Statistical analysis

Statistical analyses were performed using SPSS (version 13.0). All values are expressed as the mean ± standard deviation (SD). Paired Student’s *t*-tests were used to determine the statistical significance of pairwise comparisons. Cell proliferation data were analyzed using two-tailed *t* tests. One way analysis of variance(ANOVA) followed by Student–Newman–Keuls (SNK)-q test was used to compare data from luciferase reporter assays, real-time PCR, cell cycle assays, invasion/migration assays, TUNEL assays, growth rates for LSCC xenografts and western blots. P values less than 0.05 were considered significant.

## Results

### Levels of miR-101 in LSCC tissues and cell lines

**Fig. 1:**
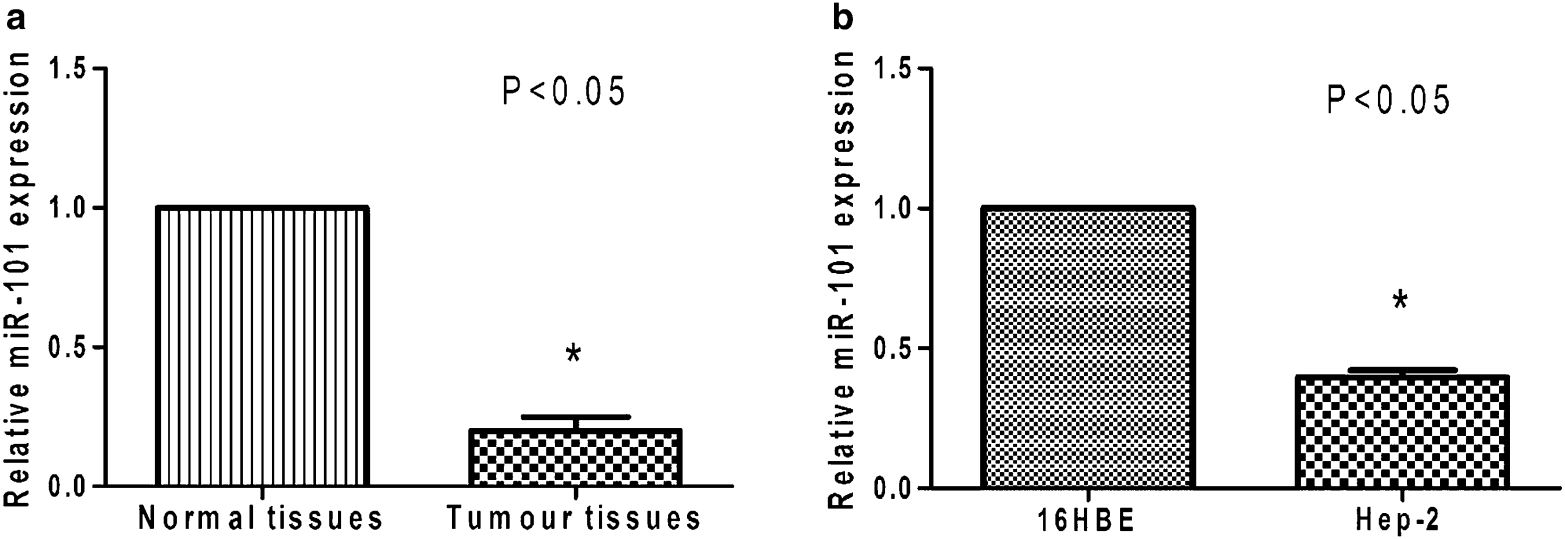
Expression of miR-101 in vivo and in vitro. **a** Levels of miR-101 that were detected in LSCC tissues were significantly lower than the levels of miR-101 detected in the corresponding adjacent, non-cancerous tissues. **b** Levels of miR-101 in the Hep-2 cells were significantly lower than in the 16HBE cells. *P < 0.05.

Levels of miR-101 were ~fivefold higher in adjacent normal tissues than in cancer tissue (from the 80 patients) (P < 0.05) (Fig. 1a). Similarly, higher level of miR-101 was detected in the 16HBE cell line compared with the human LSCC cell line, Hep-2 (P < 0.05) (Fig. 1b). We also found no significant correlation between the expression of miR-101 and the tested clinicopathological parameters, which included the patient’s sex, age, differentiation and tobacco exposure. However, the low miR-101 expression was found to be correlated with higher-grade tumours, lymph node metastases, or more advanced clinical stages of the gastric LSCC samples (P < 0.05, Table 1).

**Table 1:** Relationship between miR-101 expression levels and clinicopathological parameters for the present LSCC cohort

Clinicopathological parameters	N (No. of patients)	Level of miR-101 expression (T/N ratio)^a^	*P* value
Patient gender			0.776
Male	56	0.208 ± 0.056	
Female	24	0.212 ± 0.059	
T classification			0.015
T1–2	40	0.225 ± 0.054	
T3–4	40	0.194 ± 0.055	
Lymph node metastasis			0.044
Negative	45	0.221 ± 0.051	
Positive	35	0.195 ± 0.06	
Differentiation			0.096
Well	54	0.202 ± 0.053	
Moderately/poorly	26	0.225 ± 0.061	
Patient age			0.051
≥60 years	48	0.212 ± 0.050	
<60 years	32	0.206 ± 0.065	
Clinical stage			0.004
I–II	38	0.228 ± 0.054	
III–IV	42	0.192 ± 0.053	
Tobacco exposure			0.638
Smoker	60	0.208 ± 0.060	
Nonsmoker	20	0.215 ± 0.045	
miR-101 levels			<0.01
Low	40	0.166 ± 0.030	
High	40	0.253 ± 0.039	

^a^Tumour/normal (T/N) ratio: fold change in miR-101 expression in LSCC tissue to the corresponding adjacent normal tissue. MiR-101 expression was measured by real-time PCR and was normalized to an external control (human U6 gene). Values were quantified using the 2^−ΔΔCt^ method. Values are presented as the mean ± SD.

### Prognostic significance of miR-101 expression levels

**Fig. 2:**
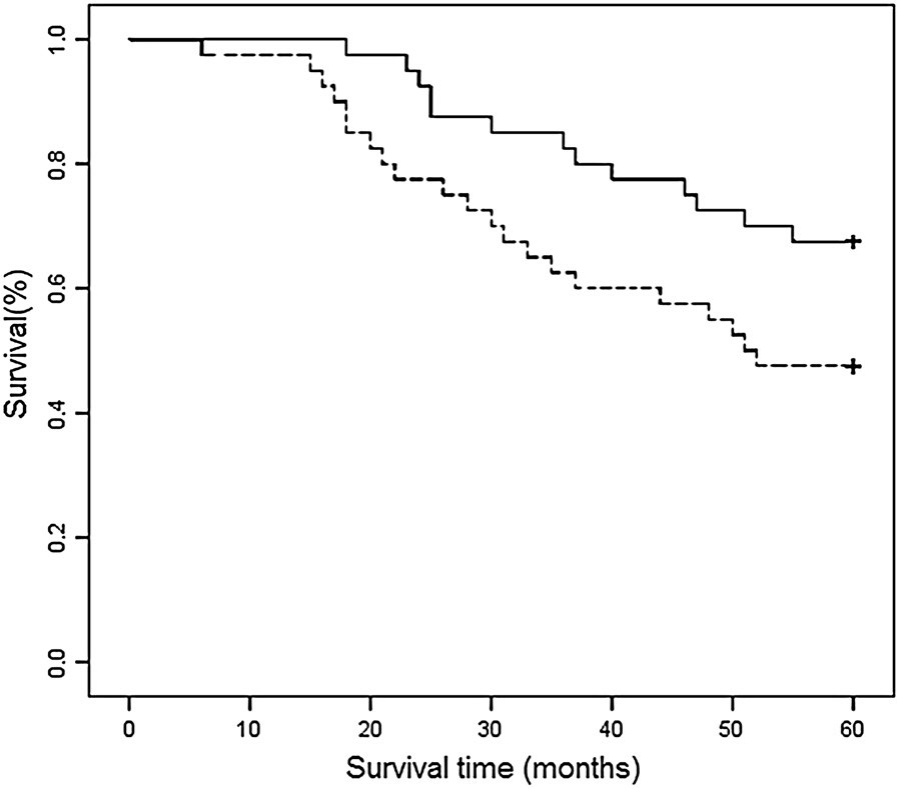
Kaplan–Meier overall survival (OS) curves for the LSCC cohort studied (n = 80). A significantly shorter 5-year OS rate was observed for patients with LSCC characterized by low levels of miR-101 expression (47.5 %; n = 40; *lower curve*) compared with LSCC patients with high levels of miR-101 (67.5 % %; n = 40; *upper curve*) (P = 0.047).

There were 80 patients that were examined and 34 died during the follow-up period. The corresponding 5-year survival probability rates for these patients were 67.5 and 47.5 % according to the expression levels of miRNA-101 (e.g., high versus low, respectively) in the LSCC tissues examined (P < 0.05) (Fig. 2). These results suggest that patients with LSCC tumours that express lower levels of miR-101 will have a poor prognosis and a shorter survival period compared with patients with LSCC tumours that express higher levels of miR-101.

### The 3′ UTR of *CDK8* is a target for miR-101

**Fig. 3:**
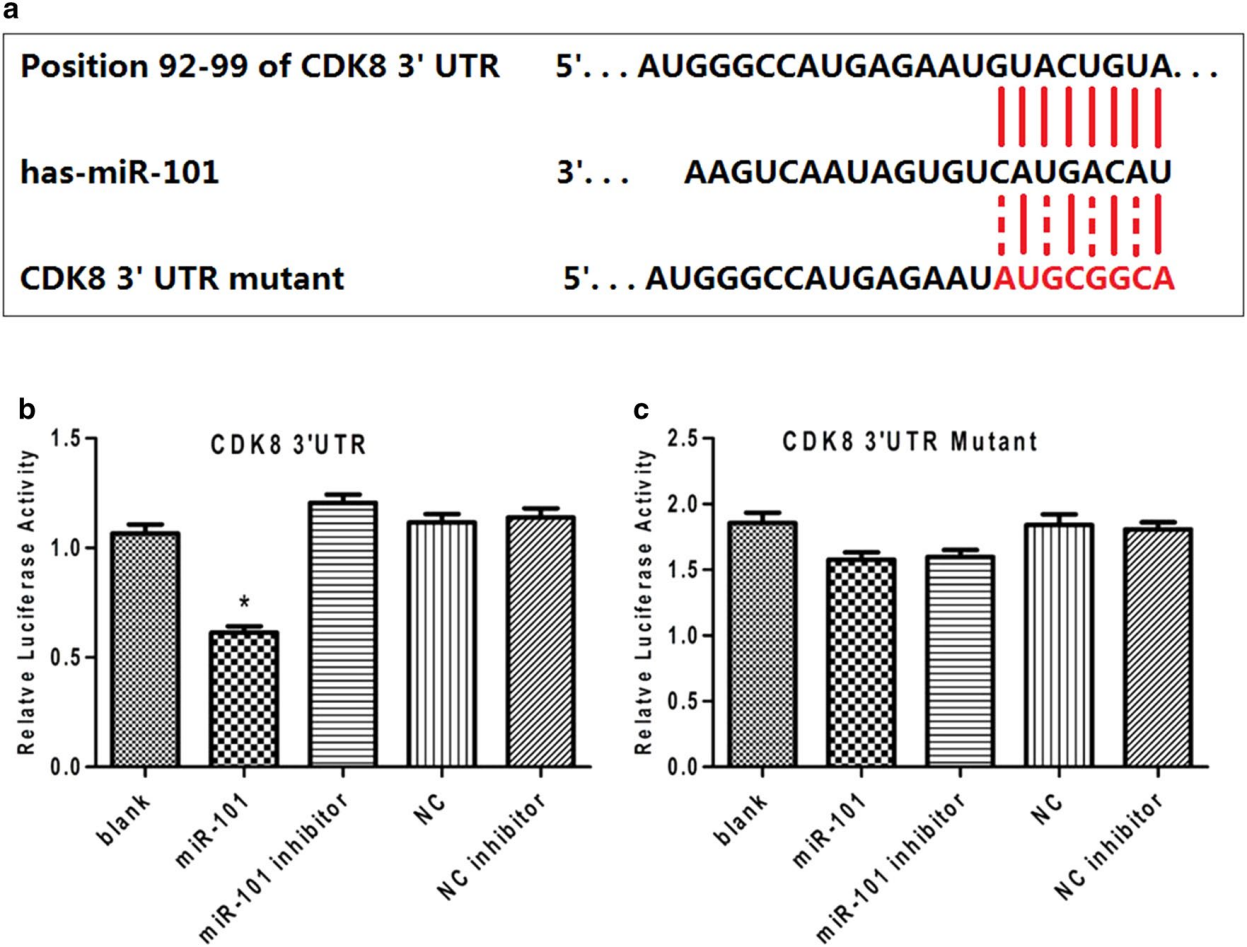
MiR-101 directly targets the 3′ UTR of *CDK8* mRNA. **a** Wildtype and mutated miR-101 target sites in the 3′ UTR of *CDK8* were cloned into luciferase reporter vectors. **b** Luciferase activity for the wildtype CDK8 reporter significantly decreased by 43 % in HEK293T cells that expressed miR-101 compared with the control cells (*P < 0.05). **c** Luciferase reporter gene assay for measuring interactions between miR-101 and 3′-UTR of the CDK8 mutant in HEK293T cells. There was no significant difference between groups (P > 0.05). The luciferase experiments were repeated three times.

To test the hypothesis that the 3′ UTR of *CDK8* is a functional target of miR-101, the miR-101 seed sequence of the 3′ UTR of *CDK8* was cloned into a luciferase reporter construct immediately downstream of the luciferase gene (Fig. 3a). In parallel, another reporter construct was generated which included a mutated version of the conserved targeting region of miR-101 within the 3′-UTR of *CDK8* (Fig. 3a). The relative luciferase activity of the reporter containing the wildtype 3′ UTR of CDK8 was significantly suppressed when miR-101 was co-transfected (Fig. 3b). In contrast, the luciferase activity of the mutant reporter was unaffected by the simultaneous transfection of miR-101 (Fig. 3c).

### Up-regulation of miR-101 in Hep-2 cells reduces cell proliferation and induces cell cycle arrest

**Fig. 4:**
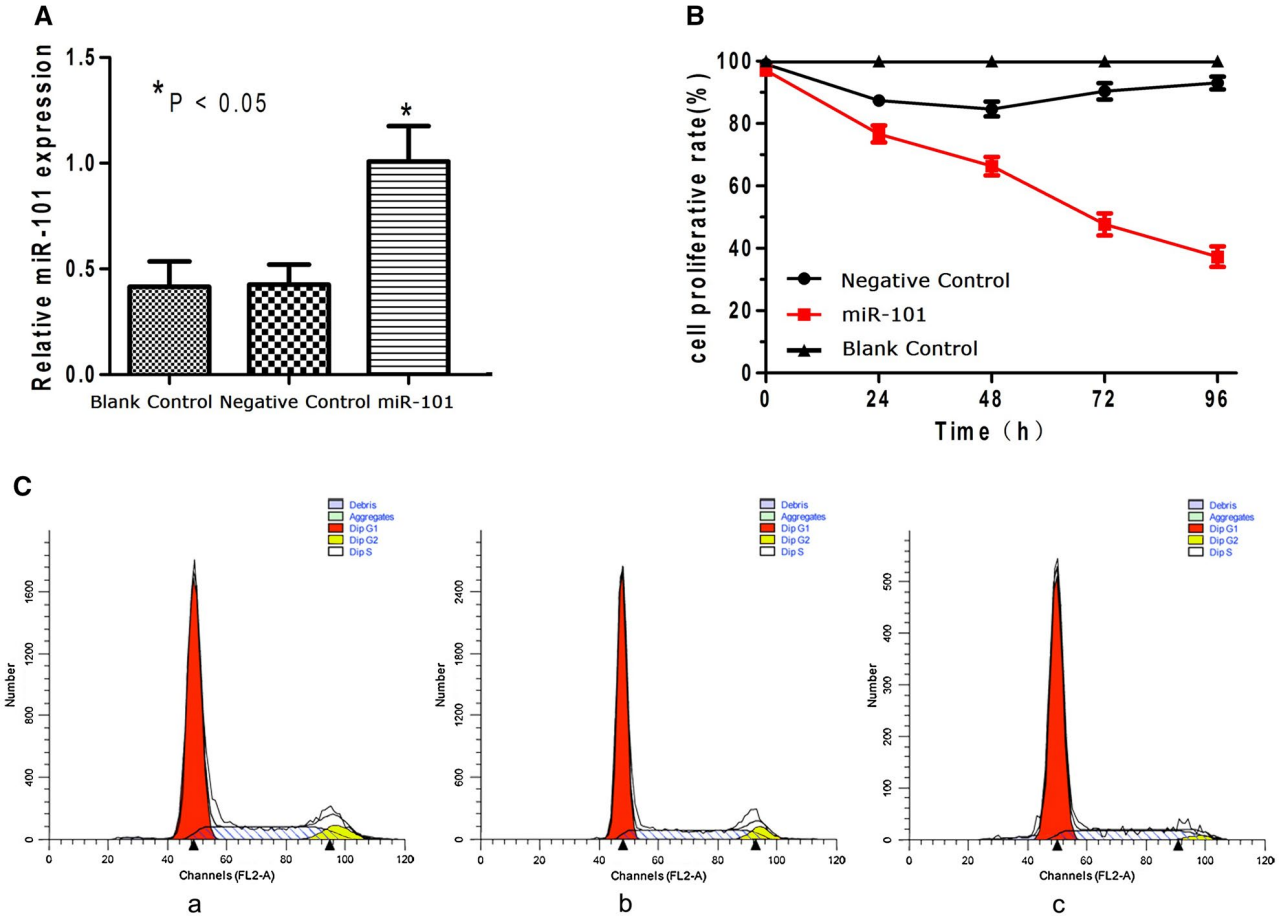
Exogenous expression of miR-101 reduces cell proliferation and induces cell cycle arrest in Hep-2 cells. **A** MiR-101 expression significantly increased in the miR-101-treated group compared with that in the negative control group and the blank control group in real-time RT-PCR assays (* P < 0.05). **B** Cell proliferation was reduced in the miR-101-treated group compared with the control groups 48, 72, and 96 h post-transduction (P < 0.05). **C** Cell cycle profiles are shown for: Hep-2 cells in the blank control group (**a**) [G0/G1 = (64 ± 2.16) %, S = (28.94 ± 2.30) %, G2/M = (7.06 ± 1.40) %]; Hep-2 cells in the negative control group (**b**) [G0/G1 = (65.11 ± 1.05) %, S = (28.53 ± 1.14) %, G2/M = (6.36 ± 1.33) %]; and Hep-2 cells in the miR-101-treated group (**c**) [G0/G1 = (73.79 ± 2.45) %, S = (22.73 ± 1.05) %, G2/M = (3.47 ± 2.27) %].

To investigate the biological function of miR-101 in LSCC, recombinant lentiviruses containing the human sequence of miR-101, as well as a GFP cassette, were generated to restore expression of miR-101 to a LSCC cell line. Seventy-two hours after transduction, greater than 80 % of Hep-2 cells in each set of the miR-101-treated group and the negative control group were found to express GFP, a marker of the infection efficiency of the lentivirus vectors (Additional file 2: Fig. S2 A-D). Correspondingly, the expression level of miR-101 was up-regulated in the miR-101-treated cells compared with that in the negative control cells and the blank control cells, as measured by quantitative real-time PCR (P < 0.05) (Additional file 2: Fig. S2 E). Proliferation of the Hep-2 cells was also measured following the transduction of lentivirus vectors. At various timepoints post- transduction (0, 24, 48, 72, and 96 h), the Hep-2 cells infected with the miR-101 lentivirus exhibited significantly less proliferation compared with the negative control cells (P < 0.05; Fig. 4B). To investigate the mechanism mediating this antiproliferative effect, a cell cycle analysis was performed. The Hep-2 cells infected with the miR-101 lentivirus showed an 8 % increase in the number of cells in the G0/G1 phase compared with the negative control cells (Fig. 4C).

### MiR-101 reduces invasion of Hep-2 cells

**Fig. 5:**
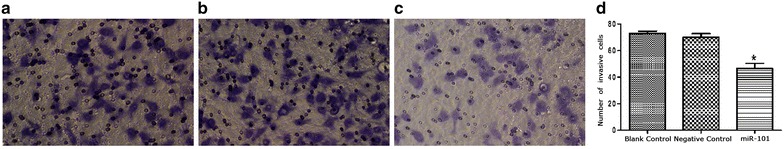
Exogenous expression of miR-101 reduces the migration of Hep-2 cells. Cell migration assays were performed 72 h after transduction using Boyden chambers coated with Matrigel. Fewer Hep-2 cells in the miR-101-treated group (**c**) migrated to the lower chambers of the transwell plates compared with the Hep-2 cells in the blank control group (**a**) and the Hep-2 cells in the negative control group (**b**). **d** Quantitation of the migration data ± SD is shown (*P < 0.05).

To investigate the effect of miR-101 on the invasive phenotype of Hep-2 cells, invasion assays were performed using 24-well Boyden chambers coated with Matrigel. Fewer Hep-2 cells infected with the miR-101 lentivirus migrated through the porous transwells 72 h after transduction (46.53 ± 6.71) compared with the negative control cells (70.07 ± 4.56) and the blank control cells (72.86 ± 6.36) (P < 0.05; Fig. 5). These data strongly suggest that miR-101 negatively affects the invasive phenotype of LSCC cells.

### MiR-101 enhances the apoptosis of LSCC cells

**Fig. 6:**
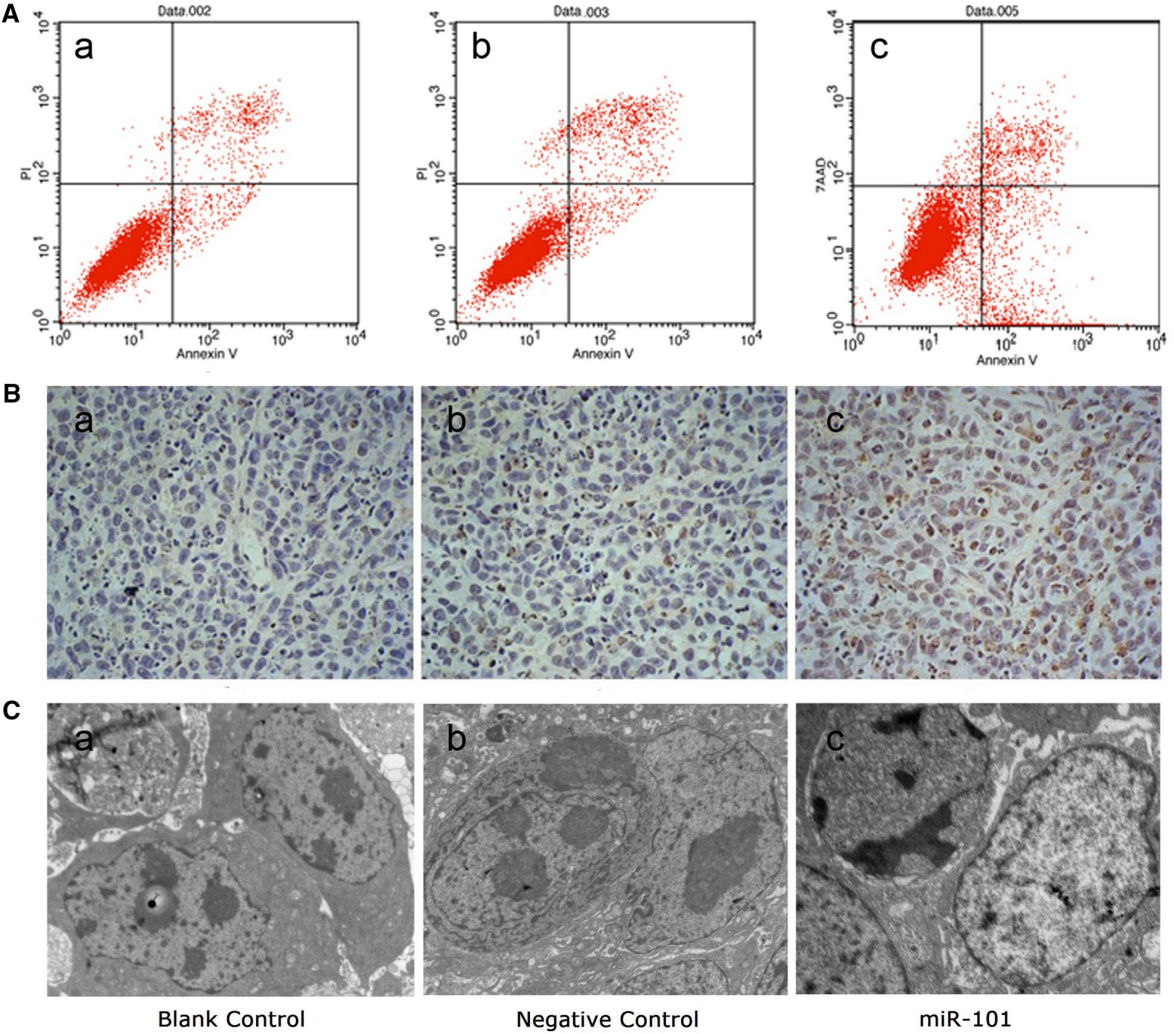
Exogenous expression of MiR-101 enhances apoptosis by LSCC cells. **A** The Hep-2 cells in the miR-101-treated group (**c**) exhibited significantly higher levels of apoptosis (12.51 ± 2.22 %) 72 h after transduction than the Hep-2 cells in the blank control group (**a**) (4.12 ± 0.85 %) and the cells in the negative control group (**b**) (3.45 ± 1.39 %) (P < 0.05). Representative histograms of the three independent experiments that were performed are shown. **B** In the TUNEL assays performed, a significantly higher number of apoptotic cells were detected in the miR-101-treated group (**c**) (27.07 ± 3.66 %) compared with the negative control group (**b**) (5.73 ± 2.43 %) and the blank control group (**a**) (4.97 ± 2.14 %) (P < 0.05). **C** Using transmission electron microscopy, tumour cells in the miR-101-treated group (**c**) were found to exhibit a morphology characteristic of apoptosis, while tumour cells in the negative control group (**b**) and the blank control group exhibited normal morphology (**a**) (×12,000 magnification).

As shown in Fig. 6A, the Hep-2 cells infected with the miR-101 lentivirus exhibited significantly higher levels of apoptosis (12.51 ± 2.22 %) 72 h after transduction than the blank control cells (4.12 ± 0.85 %) and the negative control cells (3.45 ± 1.39 %) (P < 0.05). Apoptosis was also detected for the xenograft tumour model established in vivo. For these studies, TUNEL assays were performed and significantly greater numbers of apoptotic cells were detected in the miR-101-treated group (27.07 ± 3.66 %) compared with the negative control group (5.73 ± 2.43 %) and blank control group (4.97 ± 2.14 %) (P < 0.05; Fig. 6B). Furthermore, typical signs of apoptosis, such as nuclear condensation and fragmentation, marginalization of chromatin, cell shrinkage, and formation of cytoplasmic vacuoles, were associated with the miR-101-treated tumours (Fig. 6C, c). In contrast, tumour cells from the blank control group and the negative control group exhibited healthy characteristics such as complete cellular structure, large and obvious nucleus, abundant chromatin, a mass of nuclear divisions, dual- nucleus or multi- nucleus phenomenons (Fig. 6C, a, b). Taken together, these data strongly indicate that miR-101 induces apoptosis in LSCC cells.

### MiR-101 suppresses the growth of LSCC tumour xenografts in nude mice

**Fig. 7:**
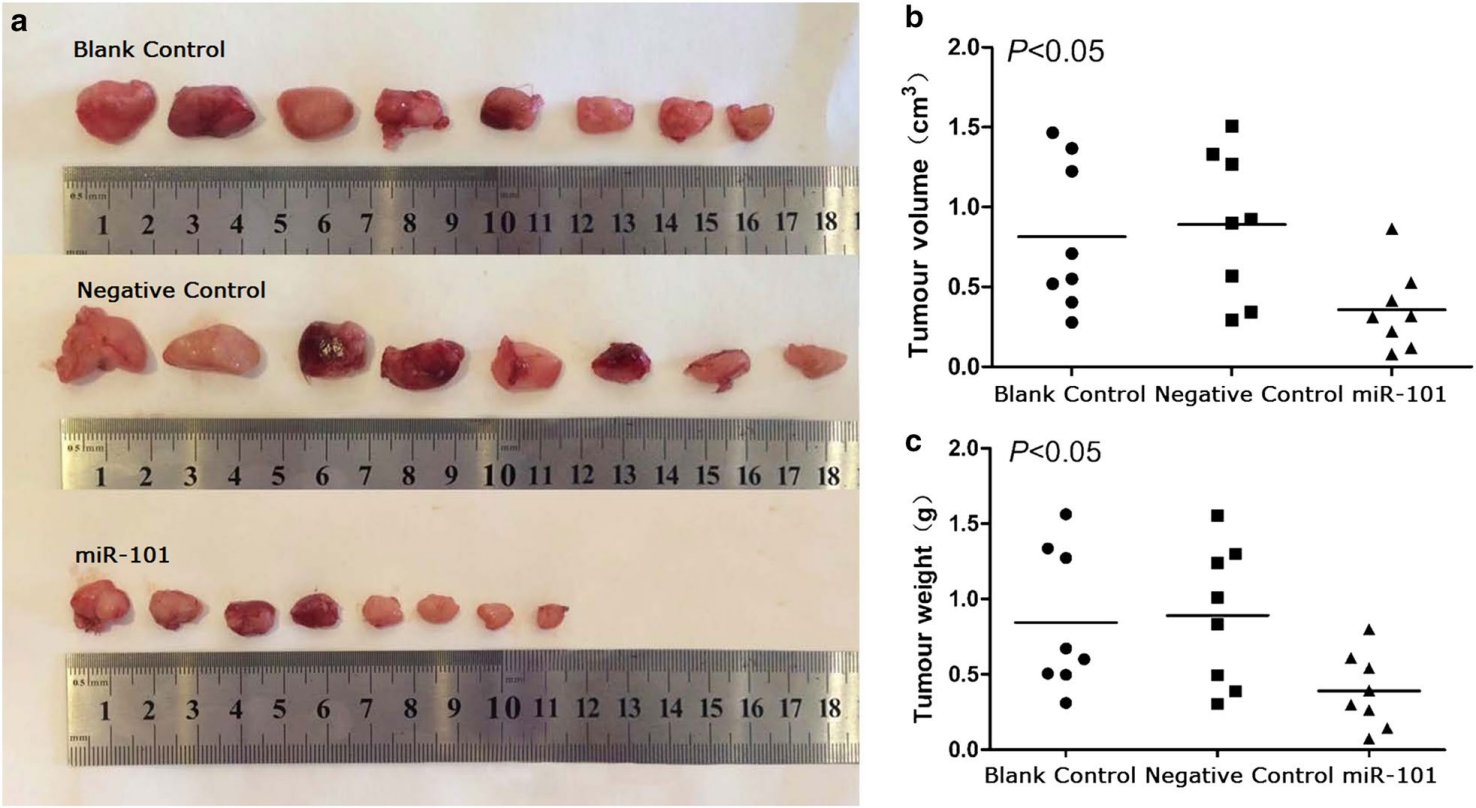
MiR-101 inhibits the growth of LSCC in a xenograft tumour model. **a** The images of xenograft tumours in different treated groups. **b** Tumours in the miR-101-treated group had a smaller volume (cm^3^) than the tumours grown in the blank control group and the negative control group (P < 0.05). **c** The average tumour weight (g) for the miR-101-treated group was also lower than that for the blank control group and the negative control group.

Twenty-four mice were divided into three groups to establish xenograft tumour models. All of the mice formed detectable tumours during the experimental period. However, the mean tumour volume for the mice treated with miR-101 lentivirus (0.34 ± 0.23 cm^3^) was much smaller than the tumour volumes recorded for the negative control group (0.82 ± 0.47 cm^3^) and the blank control group (0.89 ± 0.46 cm^3^). Similarly, the mean tumour weight for the mice treated with miR-101 lentivirus (0.41 ± 0.26 g) was markedly lower than the mean tumour weights for the negative control group (0.81 ± 0.50 g) and the blank control group (0.90 ± 0.51 g) (P < 0.05; Fig. 7).

### MiR-101 suppresses CDK8, beta-catenin, and cyclin D1 expression both in vitro and in vivo

The results of bioinformatic tools for miRNA target screening and luciferase assay indicated that miR-101 interacted with CDK8. In addition, CDK8 has been found to affect the activity of β-catenin in human colon cancer [40–42]. As a result, we interested in whether miR-101 could participate in the regulation of the Wnt/β-catenin signaling pathway through targetting CDK8 in LSCC. The expression levels of CDK8, β-catenin and downstream signalling molecules, cyclin D1, were measured in vitro and in vivo.

**Fig. 8:**
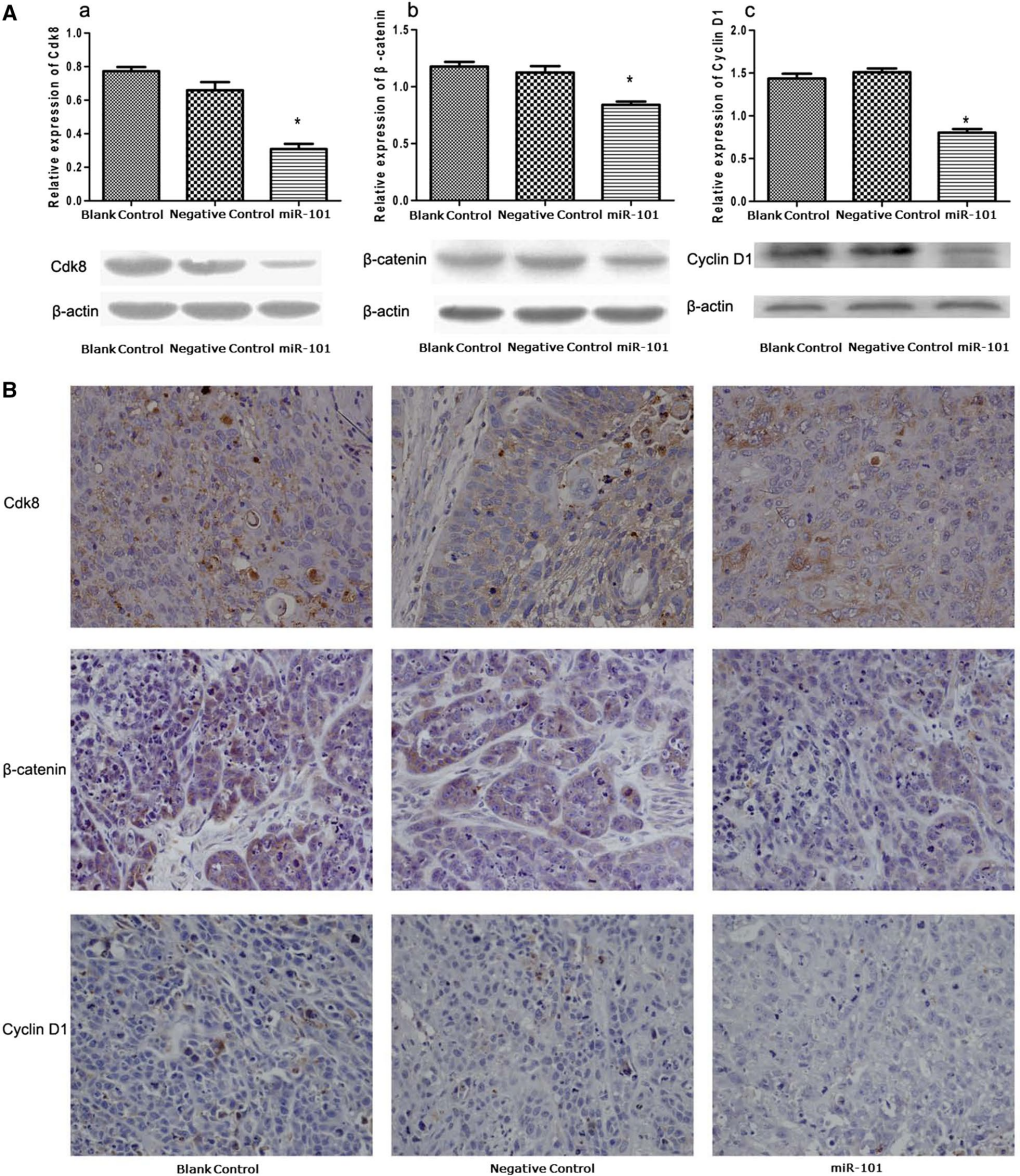
Western blotting and immunohistochemical detection of CDK8, β-catenin, and cyclin D1. **a** Expression of CDK8, β-catenin, and cyclin D1 in the cells of the miR-101-treated group were lower than the expression levels of these three proteins in cells of the blank control group and the negative control group (*P < 0.05). Detection of β-actin was used as a loading control. **b** Cytoplasmic labelling of CDK8, β-catenin, and cyclin D1 in immunohistochemistry assays were weaker in tissues of the miR-101-treated group compared with the cells of the blank control group and the negative control group.

In vitro western blotting experiments, lower levels of all three proteins were detected in the miR-101-treated cells compared with the negative control and the blank control cells (P < 0.05) (Fig. 8A). Furthermore, the expression levels of CDK8, β-catenin, and cyclin D1 were the same for the latter two cell groups (P > 0.05).

Immunohistochemical staining of tumour tissue sections was also performed to detect expression of CDK8, β-catenin, and cyclin D1 in vivo. Increased expression of all three proteins was observed in the tumours resected from the negative control mice and the blank control mice. In contrast, tumour sections from the miR-101-treated mice exhibited only weak expression of cytoplasmic CDK8, β-catenin, and cyclin D1 (Fig. 8B).

## Discussion

Accumulating evidence shows that alterations in miRNA expression levels can affect cell physiology and tumourigenesis in LSCC. For example, miR-129-5p has an oncogenic role in LSCC and directly inhibits the tumour suppressor APC [19]. Consequently, miR-129-5p has been identified as a potential target for therapeutic intervention in LSCC. Moreover, miR-203 is a tumour suppressor identified in LSCC, and it is hypothesized to regulate ASAP1 in relation to epithelial–mesenchymal transition (EMT) and cancer stem cells [46]. These findings demonstrate that miRNAs can have multiple roles in LSCC, and the mechanistic details of these roles in relation to tumour progression remain to be determined.

Aberrant expression of miR-101 has been observed in several cancer cell lines and cancer tissues [23–34]. However, little is known about its pathophysiological roles versus its roles in the carcinogenesis of LSCC. In the present study, down-regulation of miR-101 was detected in LSCC tissues and not in matching normal tissues. Furthermore, exogenous expression of miR-101 led to a decrease in cell proliferation, reduced invasion of Hep-2 cell lines, and reduced growth of a xenograft tumour in vivo. To our knowledge, these data demonstrate, for the first time, that miR-101 functions as a tumour suppressor in LSCC. Kaplan–Meier OS curves further showed that lower levels of miR-101 expression were associated with a shorter progression-free survival trend for patients with LSCC. Our results suggest that there is a potential role for miR-101 in the molecular pathogenesis, clinical progression and prognosis of LSCC.

The TargetScan and miRDB sequence analysis predicted that the 3′ UTR of *CDK8* mRNA represented a target of miR-101. In the present study, it was confirmed that CDK8 is a direct target gene of miR-101 based on the use of wildtype and mutant 3′ UTR sequences of *CDK8* in luciferase reporter assays. In addition, the in vitro and in vivo models evaluated in the present study showed that exogenous expression of miR-101 decreased expression levels of CDK8 expression.

CDK8 has been shown to be a coactivator of several important transcriptional programs, including the Wnt/β-catenin pathway [40, 41], the p53 network [47, 48], the serum response network [49], and thyroid hormone-dependent transcription [50]. This function is partially mediated by the CDK8/kinase module of the Mediator complex. Mediator is a large multisubunit complex composed of 25–30 proteins that plays a central role in the regulation of RNA polymerase II (Pol II) transcribed genes. The overall structure and function of the Mediator complex is conserved among mammals, and it is composed of four distinct modules: the head, middle, tail (representing the main complex core), and a CDK8/kinase module [51–56]. Binding of the latter to the core of the Mediator complex prevents interactions between the Mediator complex and Pol II [56]. β-Catenin recruits Mediator complexes to β-catenin/T cell factor target genes in mammalian cells via its transactivation domain which interacts with the C-terminal domain of MED12 [35]. Thus, CDK8 could directly activate β-catenin-mediated transcription targets based on its role in the Mediator complex. However, there is also evidence that indicates that CDK8 can indirectly activate β-catenin-dependent transcription targets by phosphorylating E2F1, an apoptosis activator, to inhibit its function [56, 57]. In addition, CDK8 has emerged as an important regulator of cellular proliferation [58–61], cell cycle progression [62, 63], and cell differentiation [64, 65]. This regulation is partly mediated by the phosphorylation of histone H3 proteins [38, 39], the subunits of general transcription factors [66, 67], and certain transactivators [68, 69]. In the present study, the transduction of miR-101 lentivirus led to a decrease in CDK8 expression and a decrease in β-catenin expression. However, the question whether miR-101-mediated regulation of CDK8 directly or indirectly affects Wnt/β-catenin signaling in LSCC remains. To elucidate this molecular mechanism, further studies are necessary in the future.

β-catenin is an important signaling molecule of Wnt/β-catenin pathway that participates in both normal development and tumourigenesis by regulating multiple aspects of cells, such as proliferation, migration, apoptosis, and differentiation [70–72]. Previously, we demonstrated that miR-129-5p could regulate tumourigenesis progression in LSCC by regulating of the Wnt signalling pathway [19]. Interestingly, in this study, we found that exogenous miR-101 significantly decreased β-catenin protein expression and inhibited LSCC cell proliferation, invation, and induced apoptosis in vitro and in vivo. In addition, flow cytometric analysis showed that increased expression of miR-101 caused cell-cycle arrest at the G1/S border. Thus, we further investigated the effect of miR-101 on Wnt/β-catenin pathway. We found exogenous miR-101 not only could reduce β-catenin protein expression but could also concurrently decrease the Cyclin D1 level in cultured Hep-2 cells and xenograft tissues. Cyclin D1 is an important promoter of the G1-S transition during the cell cycle, and is also an important transcriptional target gene of the Wnt/β-catenin signalling pathway [73]. Therefore, our results revealed that indirect regulation of Wnt/β-catenin signalling pathway could be a potential mechanism of miR-101 inhibiting tumourigenesis progression of LSCC.

Taken together, the results of the present study support that miR-101-induced suppression of CDK8 expression down-regulates the expression of β-catenin either directly or indirectly to suppress Wnt/β-catenin signaling. As a result, decreased expression of cyclin D1 occur. In LSCC cells, this would inhibit tumour progression, and this is consistent with the reduced proliferation and migration that were observed for Hep-2 cells expressing exogenous miR-101. The ability of miR-101 to negatively regulate tumour growth and progression was further demonstrated in vivo when xenograft models of LSCC tumours exhibited slower growth following treatment with miR-101.

## Conclusion

In summary, the results of the present study verify that miR-101 is down-regulated in LSCC tumour tissues and exogenous expression of miR-101 inhibits cell proliferation, reduces cell invasion, and induces apoptosis in LSCC. The present data also suggest that miR-101 directly inhibits the expression of CDK8 and down-regulates the protein level of β-catenin, with the latter involving the Wnt/β-catenin signaling pathway and the downstream effectors, cyclin D1. Thus, miR-101 appears to be a valuable target for the diagnosis and treatment of LSCC, and further study of this miRNA is warranted.

## Additional files

Additional file 1:
** Figure S1.** The simple figure describing the construction of lentivirus. Additional file 2:
** Figure S2.** Hep-2 cells 72 h after transduction. (A) Fluorescence microscopic images of cells in the miR-101-treated group. (B) Light microscopic images of cells in the miR-101-treated group. (C) Fluorescence microscopic images of cells in the negative control group. (D) Light microscopic images of cells in the negative control group.

## Abbreviations

LSCC: laryngeal squamous cell carcinoma
MiRNAs: microRNAs
VEGF: vascular endothelial growth factor
UTR: 3′ untranslated region
DMEM: Dulbecco’s modified Eagle medium
CCK8: Cell Counting Kit 8
GFP: green fluorescent protein
FBS: fetal bovine serum
H&E: haematoxylin and eosin
PI: propidium iodide
TUNEL: terminal deoxynucleotidyl transferase dUTP nick end labelling
DAB: diaminobenzidine
TdT: deoxynucleotidyl transferase
SDS-PAGE: sodium dodecyl sulfate–polyacrylamide gel electrophoresis
PVDF: polyvinylidene fluoride
TBS: Tris-buffered saline
TBST: TBS containing 0.05 % Tween-20
EDTA: ethylene diamine tetraacetic acid
SD: standard deviation
SNK: Student–Newman–Keuls
EMT: epithelial–mesenchymal transition
APC: Adenomatous polyposis coli
Pol II: RNA polymerase II
OS: overall survival
