# Supplementary figures and images for: MicroRNA-101 is a potential prognostic indicator of laryngeal squamous cell carcinoma and modulates CDK8
Source: J Transl Med. 2015 Aug 19;13:271. doi: 10.1186/s12967-015-0626-6 (PMC4545549; doi:10.1186/s12967-015-0626-6)

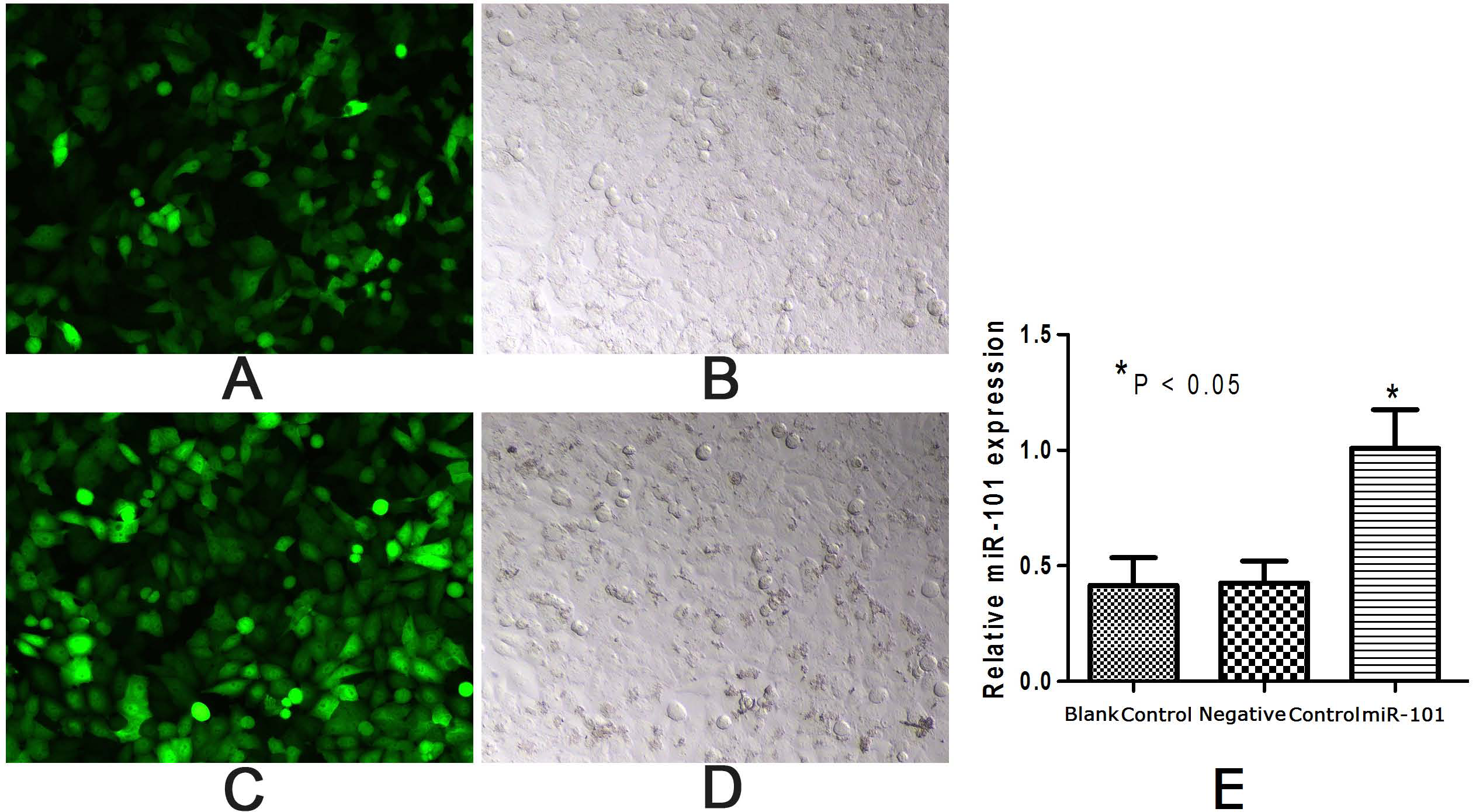

Supplement: Additional file 1: — Figure S1. The simple figure describing the construction of lentivirus. [file 12967_2015_626_MOESM2_ESM.tiff]

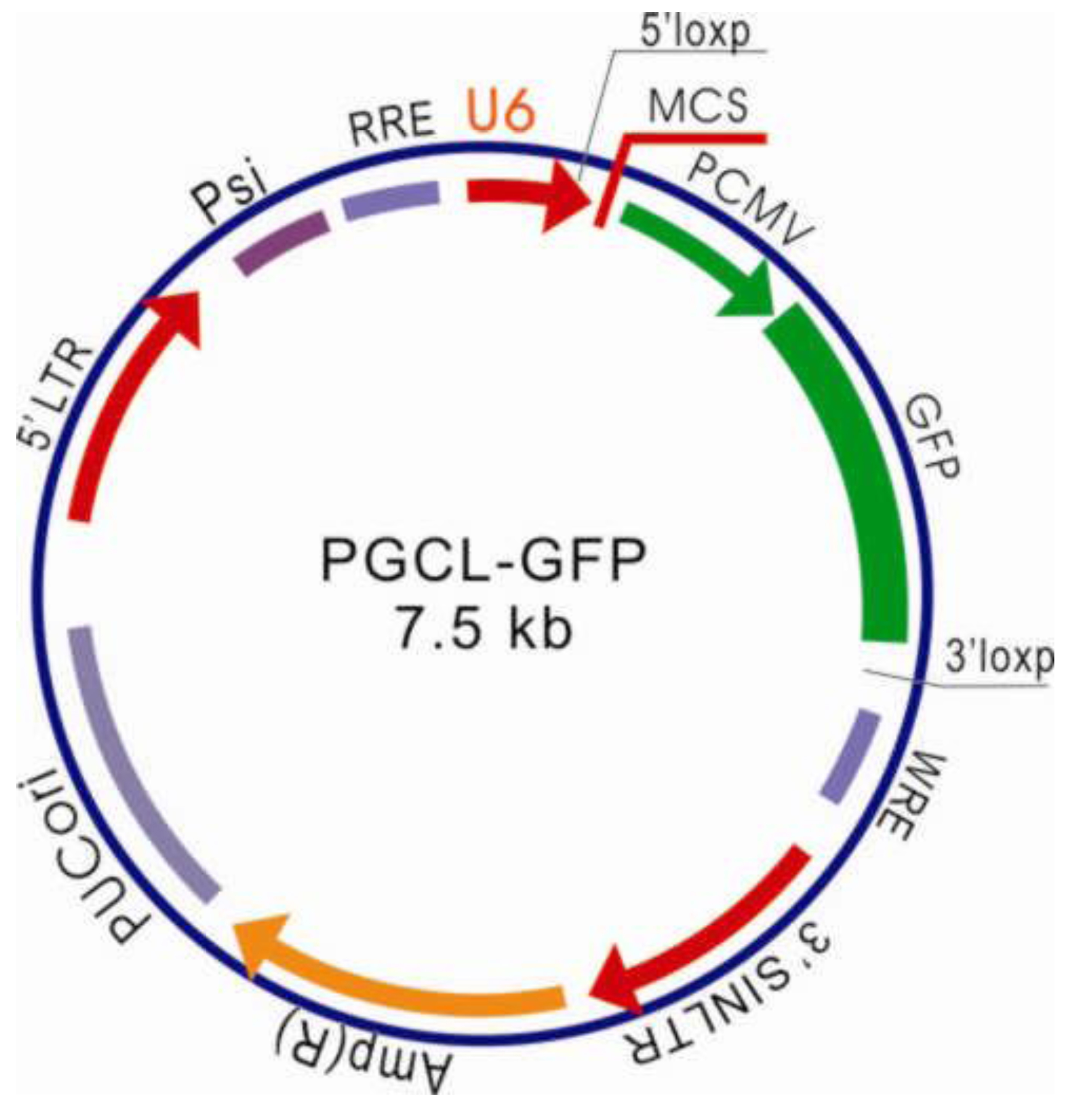

Supplement: Additional file 2: — Figure S2. Hep-2 cells 72 h after transduction. (A) Fluorescence microscopic images of cells in the miR-101-treated group. (B) Light microscopic images of cells in the miR-101-treated group. (C) Fluorescence microscopic images of cells in the negative control group. (D) Light microscopic images of cells in the negative control group. [file 12967_2015_626_MOESM1_ESM.tiff]
